# Supplementary material for: Prevalence and Predictors of Depression in Women with Osteoarthritis: Cross-Sectional Analysis of Nationally Representative Survey Data
Source: Healthcare (Basel). 2024 Feb 20;12(5):502. doi: 10.3390/healthcare12050502 (PMC10930916; doi:10.3390/healthcare12050502)
Supplement: Supplementary file 1 [file healthcare-12-00502-s001.zip › healthcare-2863181-supplementary.pdf]

Table S1. Pharmacologic Treatment for Depression

Drug Class	Generic Name	NHANES Code
SSRI	Citalopram	d04332
	Escitalopram	d04812
	Fluoxetine	d00236
	Fluvoxamine	d03804
	Paroxetine	d03157
	Sertraline	d00880
	Vilazodone	d07740
	Vortioxetine	d08125
	Nefazodone	d03808
	Trazodone	d00395
SNRI	Desvenlafaxine	d07113
	Duloxetine	d05355
	Levomilnacipran	d08114
	Milnacipran	d06635
	Venlafaxine	d03181
TCA	Amitriptyline	d00146
	Amoxapine	d00874
	Clomipramine	d00876
	Desipramine	d00145
	Doxepin	d00217
	Imipramine	d00259
	Maprotiline	d00877
	Nortriptyline	d00144
	Protriptyline	d00875
	Trimipramine	d00873
MAO-i	Isocarboxazid	d00882
	Phenelzine	d00883
	Selegiline	d00976
	Tranylcypromine	d00884
Other	Bupropion	d00181
	Mirtazapine	d04025
	Antidepressants - Unspecified	c00249

Abbreviations: Serotonin Selective Reuptake Inhibitor (SSRI), Serotonin and Norepinephrine Reuptake Inhibitor (SNRI), Tricyclic Antidepressant (TCA), Monoamine Oxidase Inhibitor (MAO-i).

Table S2. Pain Management Medications and Classes

Category/Class	NHANES Code	Formulation
Non-Steroidal Anti-inflammatory Drug (NSAID)	d04380	Celecoxib
	d00848	Diclofenac
	d04722	Diclofenac Topical
	d00208	Diflunisal
	d00851	Etodolac
	d00239	Flurbiprofen
	d00026	Fenoprofen
	d00015	Ibuprofen
	d00039	Indomethacin
	d00028	Ketoprofen
	d00273	Ketorolac
	d00283	Meclofenamate
	d00285	Mefenamic Acid
	d04532	Meloxicam
	d00310	Nabumetone
	d00019	Naproxen
	h00027	Acetaminophen; Naproxen
	d00853	Oxaprozin
	d00343	Piroxicam
	d00033	Sulindac
	d00054	Tolmetin
	c00061	Nonsteroidal Anti-Inflammatory Agents - Unspecified
Opioid	d03423	Acetaminophen; Codeine
	d03424	Aspirin; Codeine
	d00012	Codeine
	d03430	Aspirin; Caffeine; Dihydrocodeine
	d00233	Fentanyl
	d03428	Acetaminophen; Hydrocodone
	d03429	Aspirin; Hydrocodone
	d03075	Hydrocodone
	d04225	Hydrocodone; Ibuprofen
	d00255	Hydromorphone
	d00017	Meperidine
	d00050	Methadone
	d00308	Morphine
	d03431	Acetaminophen; Oxycodone
	d03432	Aspirin; Oxycodone
	d00329	Oxycodone
	d00833	Oxymorphone

	d03682	Acetaminophen; Pentazocine
	d00334	Pentazocine
	d07453	Tapentadol
	d04766	Acetaminophen; Tramadol
	d03826	Tramadol
**Other**	c00058	Analgesics - Unspecified
	c00059	Miscellaneous Analgesics - Unspecified

Table S3. Logistic Regression Results Displayed in Figure 1

	**OR (95% CI)**	**p**
**Age**		
45-54	Ref	
55-64	0.948 (0.701, 1.282)	0.725
65-79	0.669 (0.508, 0.879)	0.005
80+	0.505 (0.336, 0.76)	0.002
**Race**		
Non-Hispanic White	Ref	
Non-Hispanic Black	**0.695 (0.538, 0.899)**	0.007
Non-Hispanic Asian	**0.613 (0.426, 0.883)**	0.010
Mexican American	0.748 (0.547, 1.023)	0.069
Other Hispanic	1.21 (0.904, 1.619)	0.194
Other/Multiracial	**1.963 (1.242, 3.102)**	0.005
**Education**		
Less than 9th grade	Ref	
9th-11th	0.893 (0.622, 1.282)	0.530
HS Graduate/GED	**0.574 (0.388, 0.849)**	0.006
Some college/AA	**0.504 (0.324, 0.784)**	0.003
College graduate +	**0.334 (0.213, 0.525)**	0.000
**Marital Status**		
Married	Ref	
Widowed	**1.467 (1.07, 2.013)**	0.019
Divorced	**1.922 (1.555, 2.377)**	<0.001
Separated	1.344 (0.879, 2.055)	0.169
Never Married	**1.774 (1.243, 2.53)**	0.002
Living with Partner	1.363 (0.745, 2.493)	0.307
**Pain Medication Use**		
None	Ref	
NSAID	**2.34 (1.628, 3.362)**	<0.001
Opioid	**0.668 (0.489, 0.912)**	0.012
Both	0.886 (0.67, 1.171)	0.387
**Overweight (Y)**		
No	Ref	
Yes	**1.424 (1.227, 1.654)**	<0.001
**Health Insurance (N)**		

No	Ref	
Yes	0.606 (0.319, 1.151)	0.123
**Gap in Insurance in Past Year (Y)**		
No	Ref	
Yes	1.289 (0.918, 1.81)	0.139
**Family Income**		
>1.85 Poverty Level	Ref	
1.3< poverty level <1.85	**1.73 (1.453, 2.06)**	<0.001
Poverty Level ≤ 1.35	0.864 (0.69, 1.082)	0.197
